# Polyglutamine expansion induced dynamic misfolding of androgen receptor

**DOI:** 10.1002/pro.70154

**Published:** 2025-05-15

**Authors:** Laurens W. H. J. Heling, Vahid Sheikhhassani, Julian Ng, Morris van Vliet, Alba Jiménez‐Panizo, Andrea Alegre‐Martí, Jaie Woodard, Willeke van Roon‐Mom, Iain J. McEwan, Eva Estébanez‐Perpiñá, Alireza Mashaghi

**Affiliations:** ^1^ Medical Systems Biophysics and Bioengineering, Division of Systems Pharmacology and Pharmacy Leiden Academic Centre for Drug Research, Leiden University Leiden The Netherlands; ^2^ Laboratory for Interdisciplinary Medical Innovations Centre for Interdisciplinary Genome Research, Leiden University Leiden The Netherlands; ^3^ Department of Biochemistry and Molecular Biomedicine Institute of Biomedicine (IBUB) of the University of Barcelona (UB) Barcelona Spain; ^4^ Department of Biomedical Engineering University of Michigan Ann Arbor Michigan USA; ^5^ Department of Human Genetics Leiden University Medical Center Leiden The Netherlands; ^6^ Institute of Medical Sciences, School of Medicine, Medical Sciences and Nutrition University of Aberdeen Aberdeen Scotland

**Keywords:** androgen receptor, intrinsically disordered protein, neuromuscular disorder, polyglutamine expansion, spinal‐bulbar muscular atrophy

## Abstract

Spinal bulbar muscular atrophy (SBMA) is caused by a polyglutamine expansion (pQe) in the N‐terminal transactivation domain of the human androgen receptor (AR‐NTD), resulting in a combination of toxic gain‐ and loss‐of‐function mechanisms. The structural basis of these processes has not been resolved due to the disordered nature of the NTD, which hinders experimental analyses of its detailed conformations. Here, using extensive computational modeling, we show that AR‐NTD forms dynamic compact regions, which upon pQe re‐organize dynamically, mediated partly by direct pQ interaction with the Androgen N‐Terminal Signature (ANTS) motif. The altered dynamics of the NTD result in a perturbation of interdomain interactions, with potential implications for the binding of the receptor protein to its response element. Oligomeric aggregation of the dynamic misfolded NTD exposes pQe, but blocks tau‐5 and the FQNLF motif, which could lead to aberrant receptor transcriptional activity. These observations suggest a structural mechanism for AR dysfunction in SBMA.

## 1 INTRODUCTION

The androgen receptor (AR/NR3C4) is a crucial ligand‐activated transcription factor, which is mutated in several human pathologies (Davey & Grossmann, 2016). Spinal bulbar muscular atrophy (SBMA) (Davies et al., 2008; Parodi & Pennuto, 2011), also known as Kennedy's Disease, is a neuromuscular condition that affects approximately 1 in 40,000 men (Kennedy et al., 1968) and is characterized by the progressive loss of motor neurons in the brainstem and spinal cord, leading to muscle atrophy and weakness in bulbar and extremity muscles (Rhodes et al., 2009). The toxicity of AR in SBMA is dependent on the presence of androgens, testosterone, and its more potent derivative, dihydrotestosterone, while alterations in cellular processes ultimately lead to cell dysfunction and cell death (Lieberman et al., 2002; McCampbell et al., 2000; Mhatre et al., 1993; Morfini et al., 2006; Ranganathan et al., 2009; Szebenyi et al., 2003). The onset and progression of SBMA are associated with an expansion of the CAG trinucleotide repeat in that part of the gene that encodes the N‐terminal transactivation domain (NTD), the intrinsically disordered protein (IDP) region of AR. This longer CAG repeat is translated into an expanded polyglutamine (pQ) stretch (henceforth referred to as pQe) in the protein, but the molecular mechanisms and cellular pathways that lead to neuronal dysfunction and cell death remain poorly understood. For SBMA and other AR‐related pathologies, the underlying mechanisms remain unclear, in part, due to the limited structural models of AR.

**FIGURE 1:**
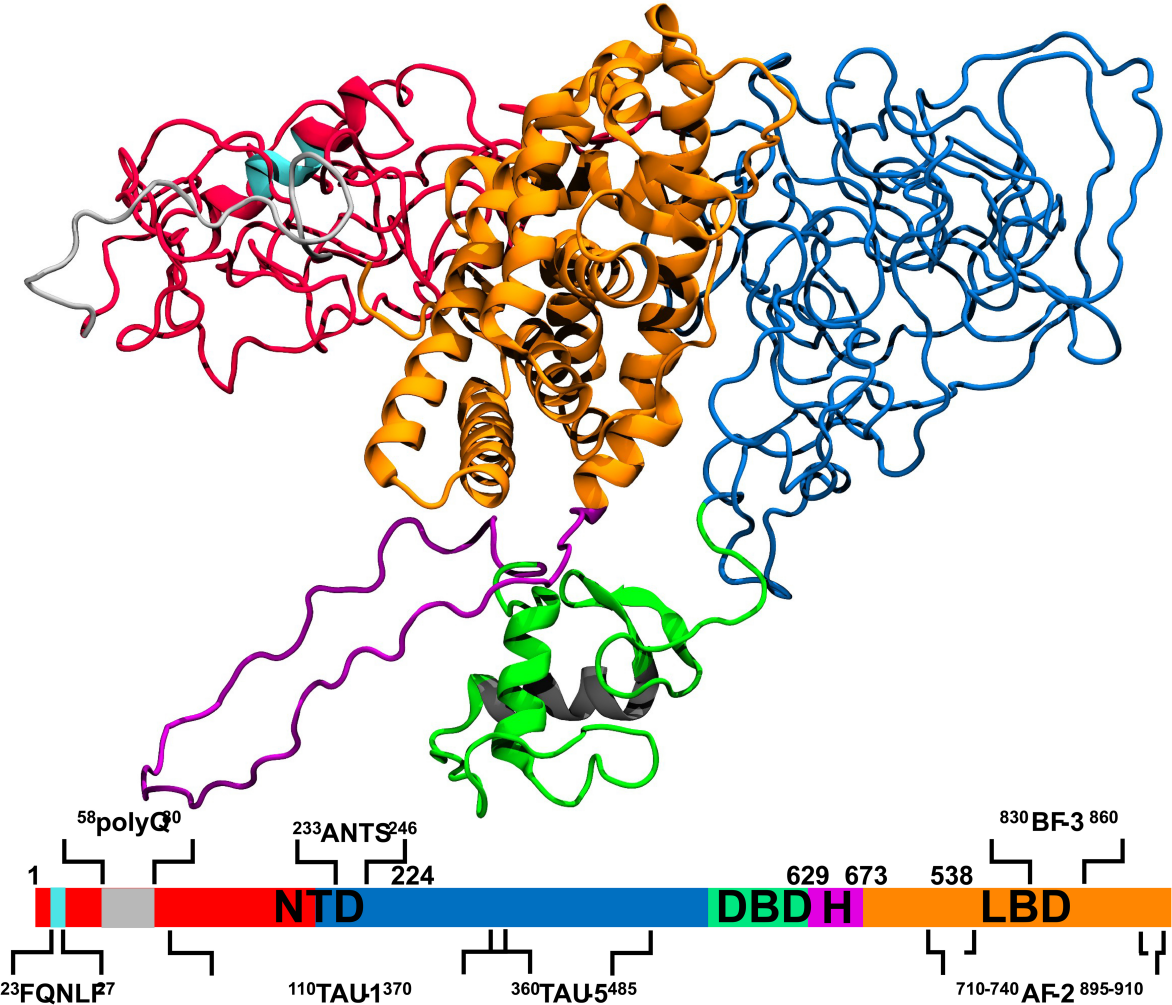
Full length model of AR‐NTD color coded by region. red: NR; blue: CR; green: DBD; purple: Hinge region and orange: LBD. The gray alpha‐helix in the DBD contains the DNA‐interacting P‐Box. Model combines the Alphafold structure of DBD‐LBD and the computationally derived wt‐NTD model published previously (Sheikhhassani et al., 2022). Model taken from Sheikhhassani et al. (2022). The graphic underneath highlights the linear structure of AR, with the subregions and corresponding residue numbers. It also contains some motifs important for AR function.

AR is a member of the nuclear receptor superfamily (Mangelsdorf et al., 1995) and displays a distinct modular structure consisting of three structural domains (Figure 1). While the ligand‐binding domain (LBD) (Bohl et al., 2005; Estébanez‐Perpiñá et al., 2005; Sack et al., 2001) and DNA‐binding domain (DBD) (Shaffer et al., 2004) have been structurally characterized to atomic level as isolated modules, a high‐resolution experimental structure of the NTD and full‐length AR remains elusive, even though low‐resolution models are available (Yu et al., 2020). In a recent computational study, we presented a first insight into a high‐resolution atomistic model of wild‐type (wt) AR‐NTD (Sheikhhassani et al., 2022). Our study revealed that the AR‐NTD forms into two spatially segregated 3D regions, the N‐terminal Region (NR) (residues 1–224) and C‐terminal Region (CR) (225–538) (Figure S1A). The overall shape and orientation of the computational model agreed well with the cryo‐EM image of the AR (Yu et al., 2020). This work provided a first 3D conformational model of the NTD, paving the way for the analysis of native and mutated forms of AR.

pQ tracts occur frequently in the human proteome, predominantly in the intrinsically disordered regions of transcription factors (Gemayel et al., 2015), yet their biological function is poorly understood (Silva et al., 2018). One hypothesis is that the pQ tracts regulate activity by altering the stability of the complexes they form (Fiumara et al., 2010) and that contractions and expansions have functional implications subject to natural selection (Gemayel et al., 2010). Variations in the length of pQ tracts have been linked to nine hereditary neurodegenerative diseases, including Huntington's disease and SBMA, colloquially known as the pQ diseases (Orr & Zoghbi, 2007). The pQ tract in AR‐NTD is also evidently involved in several other key AR pathologies. Apart from SBMA, variable lengths of this tract in AR have been linked to the onset and severity of prostate cancer (He et al., 2020; Kumar et al., 2011) and infertility (Pan et al., 2016). The structural and functional consequences of pQ expansion toward the progression of SBMA are still a matter of debate with several hypotheses proposed. Some suggest that pQe AR‐NTD is a neurotoxic species which leads to protein aggregate formation in the cytoplasm and nucleus (Adachi, 2005), while others have suggested that pQe AR‐NTD itself is neurotoxic, affecting signaling functions (Kumar et al., 2011) and autophagy (Cortes et al., 2014), possibly leading to apoptosis. AR‐NTD forms key protein–protein interactions (PPIs) with the other AR domains (DBD and LBD) as well as with over 250 different proteins to exert its function (Gottlieb et al., 2012). Through intra‐domain interactions with DBD, the NTD was previously shown to act as an allosteric regulator of DNA binding (Brodie & McEwan, 2005), while LBD interactions, also termed the N/C interaction (Schaufele et al., 2005), are seemingly important for the transcriptional activity of AR. The interdomain interactions are with an extensive range of co‐regulator proteins (De Mol et al., 2018; He et al., 2004; Ray et al., 2006; Tavassoli et al., 2010; Wafa et al., 2003). For example, RNA polymerase II‐associating protein 74 (RAP74, a subunit of the TFIIF transcription factor) promotes the transcriptional activities of AR while the C‐terminal of Heat Shock Protein (HSP)70 Interacting Protein (CHIP) mediates AR ubiquitination. While unknown, any mechanistic process through which pQe affects the interaction dynamics with these partners is plausible to contain important information regarding molecular mechanisms behind SBMA.

In this study, we employed extensive molecular dynamics (MD) simulations, topological analysis, and advanced docking approaches to model the impact of pQe in AR‐NTD (pQe: 45 glutamines, a length that has been associated with SBMA) compared to physiologically wild type (wt: 23 glutamines) present in healthy subjects. We investigated whether disease‐related pQe tracts can alter the conformational dynamics of NTD, monitored its consequences for interactions with DBD and co‐regulators, and revealed how the pQe adds to the aggregation propensity of AR in SBMA. We built a structural model that describes how the dynamic misfolding (a term we use to make a distinction with misfolding as it happens in structured proteins) of NTD leads to the gain/loss of function of AR in SBMA.

## 2 RESULTS AND DISCUSSION

### 2.1 Polyglutamine expansion alters local and global conformations and dynamics

**FIGURE 2:**
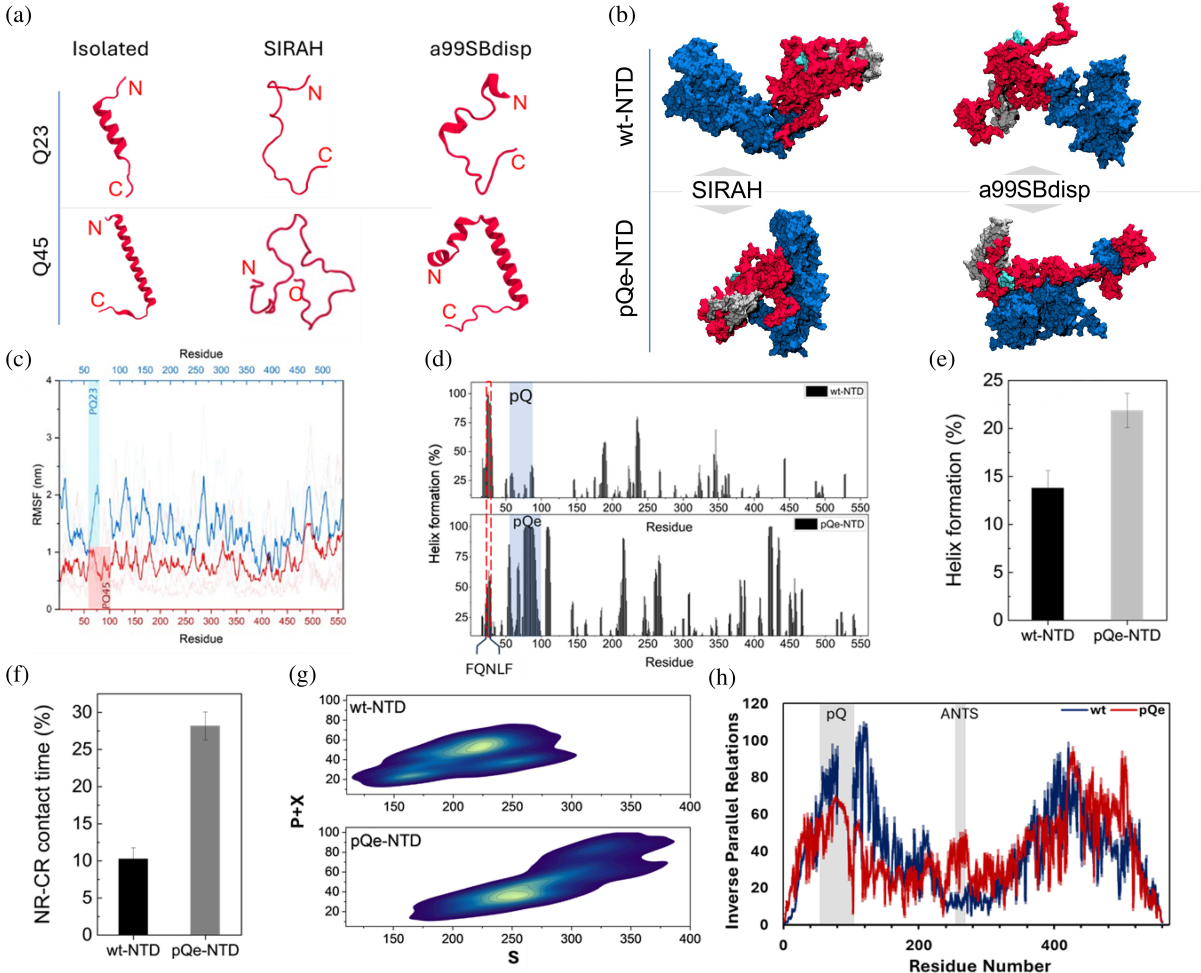
pQe results in local misfold and global alterations of protein dynamics (a) pQ tracts at the end of the simulations taken from “isolated simulations”, the SIRAH and a99SBdisp models. The results indicate that Q23 and Q45 tracts adopt different conformations. (b) pQ expansion results in an altered global conformation, both from SIRAH and a99SBdisp forcefields. NR is colored red, CR blue, the pQ region in silver and the FQNLF motif in cyan in all models. (c) Root mean squared fluctuations (RMSF) calculated per residue indicates that pQ expansion reduces chain dynamics. (d) Residue‐based α‐helix analysis of wt‐ and pQe‐NTD during the last 850 ns of all‐atom MD simulations. Residues that are involved in a stable helical formation (probability >30%) are observed in the FQNLF region and sections of the pQ and pQe tracts. (e) Quantitative secondary structure analysis reveals that pQ expansion increases the α‐helical content from 13.7 to 21.8% (error: STD over three simulations). (f) Contact time between the CR and NR regions of the wt‐NTD and pQe‐NTD. It is defined as the fraction of time during the last 850 ns of simulations when residues from the NR and CR were in contact (error: STD over three simulations). (g) Two‐dimensional density plots comparing the topological configuration space of the wt‐NTD and pQe‐NTD. Each contour plot shows the distribution of combined P + X topological relations as a function of S topological relations, aggregated over three independent simulation replicates. The contour levels represent the probability density of sampled configurations, with lighter shades indicating regions of higher probability. The axes represent the number of residues (×1000). (h) Residue‐based local circuit topology (CT) for WT and pQe AR NTD. The mean number of inverse parallel relations (±SEM, shown as shades) is shown as a function of residue number. The pQ tract and ANTS motif are highlighted by gray‐shaded areas.

We conducted all‐atom MD simulations using a99SBdisp on isolated pQ tracts to understand the effect of expansion on their structural dynamics. Initially, we simulated pQ23 and pQ45 for 500 ns and found that pQ23 adopts an alpha‐helical rod shape (Figure 2a), consistent with previous reports (Escobedo et al., 2019). Our simulations indicate that pQ45 adopts α‐helical structures with varying lengths and orientations. This aligns with observations that pQ tracts are structurally plastic, adopting conformations ranging from random coils to α‐helices, influenced by factors such as repeat length and sequence context (Bhattacharyya et al., 2006; Davies et al., 2008; Fiumara et al., 2010; Vitalis et al., 2007) Our observation highlights that flanking sequences modulate pQ structure, either stabilizing helical conformations or promoting aggregation‐prone assemblies (Davies et al., 2008; Escobedo et al., 2019; Saunders & Bottomley, 2009; Wetzel, 2012). Studies have shown that coiled‐coil domains flanking pQ tracts play a role in stabilizing aggregates, enhancing their formation (Fiumara et al., 2010). The conformational heterogeneity and helical propensity of pQ tracts further support the importance of flanking regions (Barbosa Pereira et al., 2023), which influence aggregation. These studies suggest that factors such as charged or polar residues and the propensity to form coiled‐coil structures adjacent to pQ tracts influence solubility and structural stability, thereby modulating aggregation pathways (Chiti & Dobson, 2017). The pQ repeat length has also been shown to directly influence the folding and conformational dynamics of the AR‐NTD, affecting its structural and functional state (Davies et al., 2008). These context‐dependent behaviors thus raise the question of how an expanded pQ tract impacts the global architecture and dynamicity of AR‐NTD.

To address this, we performed coarse‐grained SIRAH MD simulations on full‐length NTD with a pQe stretch, using our previously published AR modeling approach (Sheikhhassani et al., 2022). To ensure our initial conditions were independent of the results, we performed these simulations multiple times (*n* = 7) with different starting velocities. In contrast to wt‐NTD (Figure S1), we observed that within 2 μs, the expanded pQ tract forms a globular structure different from what was observed for isolated pQ45 (Figure 2a), suggesting local misfolding upon expansion influenced by flanking residues. Importantly, the pQe‐NTD forms a dynamic globular conformation whereby the segregation of the dynamic sub‐regions is less prominently observed as in wt‐NTD (Figure 2b). Furthermore, the pQ tract as well as the other physiologically important motifs, like the (Orr & Zoghbi, 2007) FQNLF (Adachi, 2005) and the KELCKAVSVSM (also known as the Androgen N‐terminal signature [ANTS] motif [Oppong et al., 2017; Shen & Coetzee, 2005]: UniProt residues 233–246, pQe‐NTD residues 255–268) remain surface exposed, despite the drastic architectural changes. We continued the simulations a further 3 μs (for all 7 runs) to follow its dynamicity over a longer time. The molecule showed high levels of structural dynamics, yet the dynamicity of pQe‐NTD was reduced when compared to wt‐NTD (Figure 2c); however, it still maintained a larger root‐mean‐square fluctuation (RMSF) when compared to a folded protein, like AR‐LBD (Kumar, 2012).

Given the importance of our observations, we decided to validate the conformational patterns of the NTD using highly accurate but computationally expensive all‐atom simulations using a99SBdisp, a force field which is tailored to IDP modeling (Robustelli et al., 2018). To mitigate potential bias introduced by initial structure assumptions, we initiated these all‐atom simulations from fully extended conformations for both stretched wt‐ and pQe‐NTD. The all‐atom simulations corroborated the dynamic, compact conformations observed in the coarse‐grained simulations, particularly the distinct segregation of the N‐terminal and C‐terminal regions in wt‐NTD (Figure S1). Notably, the consistent absence of inter‐regional contacts between these domains across both simulation methodologies (Figure S2) reinforces the robustness of our observed structural dynamics. While both simulation approaches effectively captured the overall dynamic nature of the NTD, we observed differences in the representation of secondary structure, specifically regarding alpha‐helix formation within the NR. Quantitatively, the all‐atom simulations revealed a significantly higher percentage of alpha‐helical content (13.3% ± 2.1%) compared to the coarse‐grained simulations (6.6% ± 0.6%). This discrepancy is consistent with the established accuracy of the a99SBdisp force field in modeling helical structures, a critical feature for IDP dynamics. Remarkably, the 13.3% helical content observed in our all‐atom simulations is consistent with circular dichroism results, which reported an alpha‐helical content of 14% (Davies et al., 2008), providing compelling experimental validation for our computational findings.

Upon pQ expansion, the NR dynamically misfolds and interacts with the CR (Figure 2b and S1). This is reflected by an increased contact time between NR and CR (Figure 2f). In addition, as a result of the global restructuring caused by pQ expansion, the α‐helical content in NTD increased by >8% as determined through structural analysis quantification (Figure 2d,e) and comparable to experimental observations (Davies et al., 2008).

To understand the role of the glutamine residues in these altered fold dynamics, we mapped its interaction network with other NTD residues. It shows that the pQe tract formed longer‐range contacts, including with residues in the CR region, a behavior not observed in wt‐NTD (Figure S2). These long‐range contacts also included directional hydrogen bonds, which likely explain the lack of regional segregation and reduced dynamicity in pQe. The CR residues that the pQ tract interacts with partially overlap with the ANTS motif (Figure S3) (Oppong et al., 2017).

To quantify the structural differences between wt and pQe AR NTD, we sought to capture key topological features of the protein chain. Unlike traditional geometric methods, topological analysis is particularly well‐suited to quantify and categorize the residual structural properties of proteins that are continuously deforming, such as IDPs. By mapping the pQe NTD dynamics onto a topological space, we identified recurring motifs and patterns within the dynamics while showing a recognizable difference with wt‐NTD (Figure 2g). To probe these differences further, we tracked the topological dynamics of the NTD over time, employing the Circuit Topology (CT) framework (Mashaghi et al., 2014; Moes et al., 2022; Scalvini et al., 2023; Woodard et al., 2022) to calculate the residue‐based parallel (P), series (S), and cross (X) topologies within the chain's conformations at 25 ns intervals (Figure S4). Residue contacts, defined by spatial proximity, serve as a fundamental topological representation of the chain, offering deep insights into the dynamics and how they are altered in pQe. This revealed that, in agreement with our contact analysis, the pQ region in wt is enriched in inverse parallel relations (Figure 2g), indicating it is more sequestered in the structure than in pQe NTD. In contrast, the ANTS motif is more sequestered in pQe NTD (Figure 2g), revealing a long‐range contact between the pQ tract and this motif in pQe NTD.

Overall, both SIRAH and a99SBdisp modeling support a model in which wt‐NTD adopts segregated dynamically compact regions. Importantly, local misfolding due to pQ expansion propagates to global reorganization in NTD, in which these segregated regions dynamically merge and unmerge. Next, we investigated the implications of the observed dynamic misfolding for inter‐domain interactions.

### 2.2 Gain of function, loss of regulation: pQ Expansion induced dynamic misfolding alters PPIs

The roles of AR span genomic and non‐genomic pathways (Davey & Grossmann, 2016), which are both tightly regulated in cells. Our previous wt‐AR‐NTD model suggested a structural mechanism for the regulation of AR‐DBD binding to DNA through competing NR and CR interactions. We showed that CR binding would cover the P‐Box, the zinc finger of the DBD that binds to the major groove of DNA and thus affects the ability of the DBD to interact with DNA (Sheikhhassani et al., 2022). These observations are consistent with previously published experimental studies, which identified the NTD and stretches of the CR as having DNA binding regulatory functions (Brodie & McEwan, 2005; He et al., 2001; Liu et al., 2003; Schaufele et al., 2005; Wasmuth et al., 2020).

**FIGURE 3:**
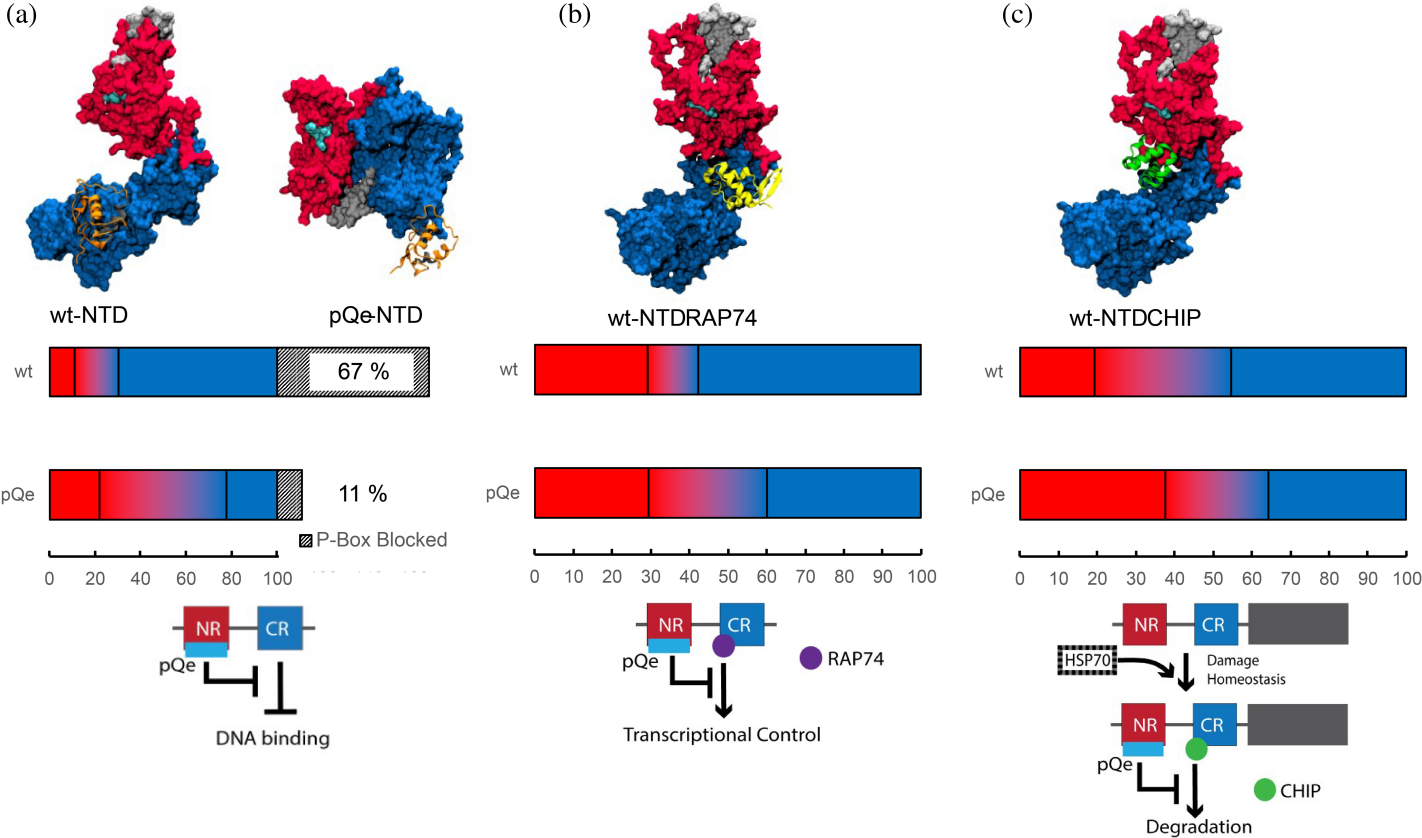
Protein–protein interactions are altered as a result of pQ expansion. (a) Representative structure of wt‐ and pQ‐NTD interacting with DBD (orange). CR‐mediated DNA binding regulation was reduced in pQe‐NTD due to exposure of P‐Box despite (black) NTD‐DBD interactions. The pQ region is colored in silver and the FQNLF motif in cyan in all models. (b) Representative structure of wt‐NTD interacting with RAP74. pQ expansion induces a change in binding sites, reducing transcriptional control. (c) Representative structure of wt‐NTD interacting with CHIP. Bar charts indicate the fraction of poses interacting with NR (red) NR/CR (dual colored) and CR (blue) residues. Graphics underneath highlight the mechanistic changes upon pQ expansion.

To evaluate the impact of pQ expansion on NTD‐DBD interactions, we conducted molecular docking using models derived from both coarse‐grained and all‐atom simulations. Initially, we clustered our simulations, selecting cluster representatives that accounted for at least 50% of the total simulation frames to ensure adequate conformational sampling. These representatives were then docked against DBD using ClusPro, generating up to 120 potential interaction poses per docking experiment (420 poses for wt‐NTD and 406 poses for pQe NTD). Given their ability to capture broader conformational sampling, essential for representing the inherent flexibility of NTD interactions, we prioritized the coarse‐grained results in our primary analysis (Figure 3). The all‐atom derived models, while providing higher atomic resolution, offer limited conformational sampling. These results are included in the supplementary information (Figures S5–S7) to provide an additional layer of validation, specifically focusing on the detailed structural aspects of the interactions.

Our findings indicate that the altered structural dynamics of pQe‐NTD significantly affect its interactions with the DBD. In the wild‐type (WT) model, the majority of identified interacting residues are localized within the CR region of the NTD. However, this interface was disrupted in the pQe variant (Figure 3a), where interactions with DBD are predominantly mediated by NR residues (Figure S6). Specifically, the ability of the NTD to block the P‐box—a critical regulatory site for DNA binding—was markedly reduced, with only 11% of docking poses achieving P‐box blockade compared to 67% in the WT model. This reduction aligns with prior reports of increased DNA binding in AR with expanded pQ tracts but contrasts with their observation of reduced AR transcriptional activity (Belikov et al., 2015).

To explore this apparent discrepancy, we used our docking approach to model interactions between pQe‐NTD and RAP74, a subunit of the TFIIF complex (Lavery & McEwan, 2008; Mcewan & Gustafsson, 1997). RAP74 plays an essential role in the formation of the pre‐initiation complex, facilitating the specificity and efficiency of RNA Polymerase II recruitment to DNA (Lei et al., 1999). Consistent with the observed DBD interactions, our model (based on 414 docking poses for pQe and 417 wt) revealed that the predominantly CR‐mediated interactions with RAP74 in pQe‐NTD were suppressed, instead having interactions covering both the NR and CR regions (Figure 3b, Figure S6) while the types of interactions suggest similar strengths as with wt‐NTD (Figure S7). These findings present a structural model illustrating how pQ expansion disrupts regulatory interactions critical to AR transcriptional activity.

## 3 NTD HAS A PROPENSITY TO AGGREGATE AS A RESULT OF ALTERED DYNAMICS BY pQ EXPANSION

**FIGURE 4:**
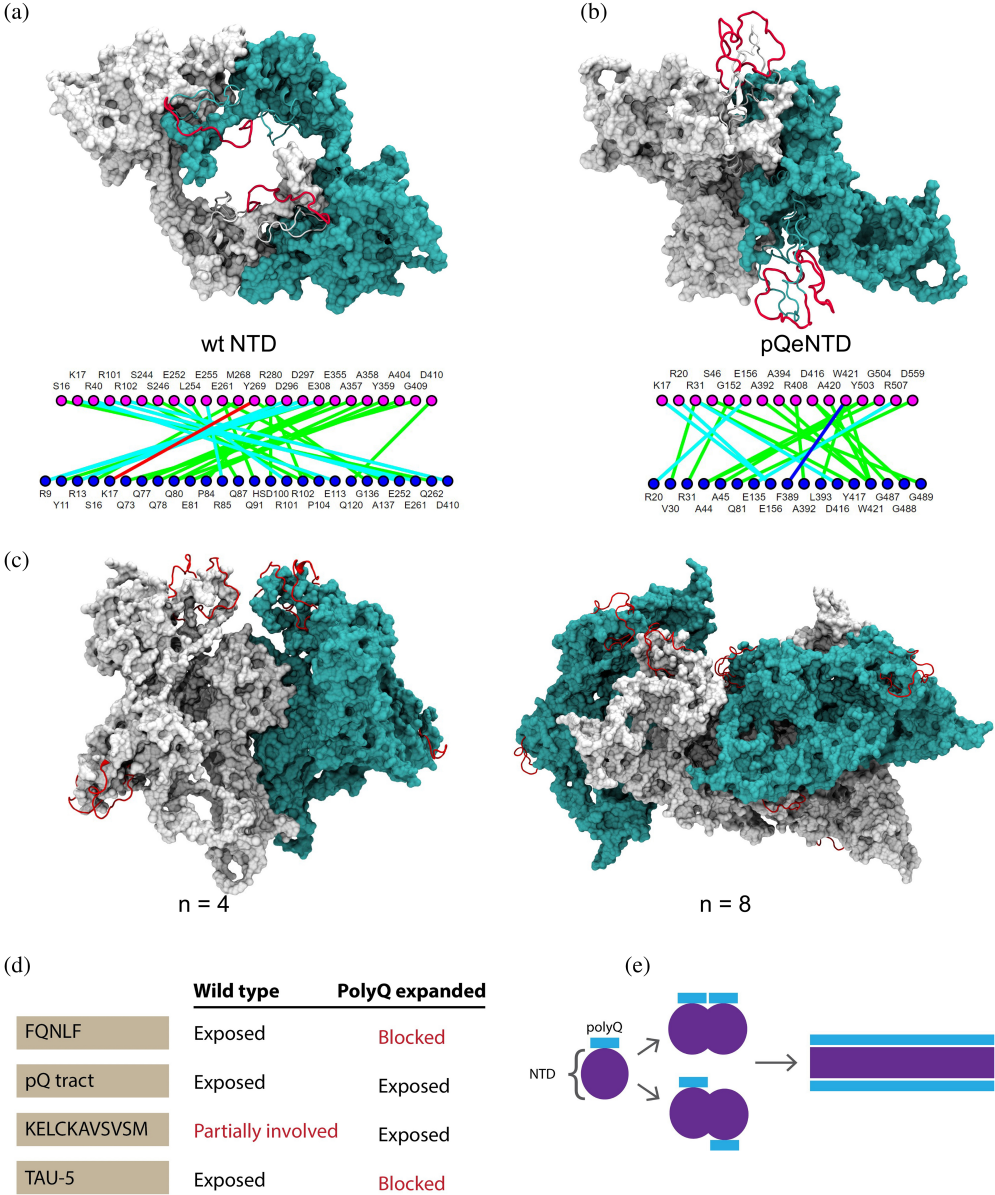
Multimerization of pQe NTD leads to fibrillar aggregates. Representative structure and residue interaction map of (a) wt‐NTD and (b) pQe‐NTD dimer. The white and cyan structures represent one NTD monomer, the pQ tract is depicted in red. The precise interactions we saw between the two monomers for wt‐ and pQe‐NTD are depicted under the representative structures. Different types of interactions are colored differently: Hydrogen bonds (green), Salt Bridge/Ionic (cyan), π‐Cation (red), and π‐Stacking (blue). (c) Models of tetramer and octamer of pQe AR‐NTD. The exposed pQ tracts are depicted in red. The cyan and white in these models each depict half the total number of proteins in the oligomer. (d) Table of functionally important motifs and their involvement in a NTD dimer. “Exposed” means that the motif is exposed on the surface of the dimer, while “blocked” means that it is buried in the interaction interface. “Partially involved” means that a subpart of the motif is involved in the dimer interface. (e) Aggregation model of pQe NTD, leading from a monomer to a fibril with the pQ tracts exposed.

Expansion of the glutamine tracts leads to ligand dependent protein aggregation in AR (Adegbuyiro et al., 2017; Chevalier‐Larsen et al., 2004; Katsuno et al., 2002; Lisberg et al., 2024; Takeyama et al., 2002; Yu et al., 2006), which in turn leads to nuclear inclusions where AR proteins get proteolysed (Heine et al., 2015). This is widely regarded as one of the pathological hallmarks of SMBA. To explore how the altered structural dynamics we have seen in our pQe‐NTD model impact oligomerization, we first performed self‐docking studies based on wt‐NTD. This dimer model (highest representative cluster of 396 docking poses) demonstrates that the two monomers adopt both asymmetric head‐to‐tail (N‐C) conformations (Figure 4a) and symmetric head‐to‐head (N‐N) conformations (Figure S8A). These dimeric poses further reveal a spatial gap between the two molecules, in a manner that is consistent with the low‐resolution cryo‐EM AR structure (Yu et al., 2020). This configuration supports the docking and interaction of the previously characterized active LBD dimer (Nadal et al., 2017) in agreement with the crystal structure of the AR‐LBD dimer.

Subsequently, we generated a pQe‐NTD dimer (from 426 poses), which illustrates a loss of the spatial gap (Figure 4b) and the conformational characteristics seen in the wt‐dimer (Figure S8B). Notably, over 50% of the docking poses of pQe‐NTD block the FQNLF motif (Figure 4b—interaction map and Figure 4d), while the pQ tract and ANTS motif remain exposed on the surface of the dimer. The FQNLF is crucial for AR activation, a process partly regulated by the competition between chaperone proteins such as HSP40 and HSP70, and the AF‐2 coactivator binding pocket. NMR data have shown that HSP70 directly interacts with FQNLF in wt‐NTD, preventing aggregation in vitro (Eftekharzadeh et al., 2019). Furthermore, modulating HSP70 was found to increase solubility, ubiquitination, and clearance of pQe‐NTD (Eftekharzadeh et al., 2019). Hsp70 cycles between ATP‐ and ADP‐bound states, with the ADP‐bound state exhibiting significantly enhanced substrate binding affinity (Mayer & Bukau, 2005). This state allows HSP70 to discriminate between properly folded and damaged proteins. The most effective HSP70 compounds stabilize it in the ADP‐bound states (Young et al., 2016), which suggests that, in the case of pQe‐AR, HSP70 could wrongly release AR‐NTD.

To explore chaperone interactions further, we examined the binding of HSP70 and CHIP with both wt‐NTD and pQe NTD. We observed that while HSP70 interacts through the region with the FQNLF in wt‐NTD, as reported in the NMR data, this interaction is absent in pQe‐NTD (Figure S9). While CHIP interacted with wt‐NTD primarily through CR residues and in the groove covering NR and CR, this interaction was significantly reduced in pQe‐NTD (Figure 3c). Instead, interactions with NR residues increased, including π‐Cation bonds (Figure S7). Together, these results suggest that in the absence of HSP70 modulation, pQe‐AR is less likely to be soluble and ubiquitinated for degradation (Figure 3c, model).

Previous reports have shown that pQe‐AR‐NTD also undergoes an altered oligomerization process compared to wt‐NTD, forming fibrils instead of annular oligomers in vitro (Jochum et al., 2012). To explore if our pQe‐NTD model could provide atomistic details to this process, we built a higher‐level oligomerization model, factoring in tetramer and octamer pQe NTD models (Figure 4c). Our results mirror those observed in dimerization, where the pQe tracts and ANTS motifs of the individual NTDs are exposed on the surface, while motifs with functional implications like the Transactivation Unit (Tau)‐5 and FQNLF mediate interactions between individual molecules. Oligomerization to an octamer demonstrates a clear tendency to form a fiber. While these specific oligomeric species (*n* = 4 and *n* = 8 AR molecules) have not been directly observed in vitro or in vivo, the protofibril model we have produced agrees with the AFM results (Jochum et al., 2012). This data allowed us to build a model of multimerization of pQe‐NTD (Figure 4e), from a monomer, via oligomers into protofibrils. This multistep oligomerization was also observed in vitro and in vivo, where pQ expansion in the NTD causes oligomerization beyond dimers via SDS‐soluble oligomers (Eftekharzadeh et al., 2019; Jochum et al., 2012; Li et al., 2007). Our model highlights that the expanded pQ tract is not the main interaction face in this oligomerization phase; rather, it remains exposed on the surface of the protein, together with the ANTS motif. This motif was previously shown to be involved in the nucleation of AR‐NTD and was suggested to be a regulator for the pQ tract (Oppong et al., 2017) in a manner comparable to the N17 domain in Htt (Cho, 2025). Our model, which further highlights the importance of these two motifs through its exposure, could signify a further step in oligomerization, where the protofibrils bind together through these exposed areas together to form stable bundles.

This model of AR‐NTD oligomerization shows similarities with studies on pQe Ataxin‐3, a protein associated with another pQe disorder, Machado–Joseph disease. In Ataxin‐3, the expansion of the pQ tract leads to misfolding and aggregation in a two‐step process (Ellisdon et al., 2006). It transitions from SDS‐soluble oligomers driven by the ordinarily structured Josephin domain (Lupton et al., 2015) with the pQ tract exposed to insoluble aggregates (Natalello et al., 2011), driven by exposed pQ‐pQ interactions (Chow et al., 2004; Ellisdon et al., 2006; Ellisdon et al., 2007). While the model we present here does not take ordinarily structured domains (AR LBD and DBD) into account, it could be that the altered structural dynamics caused by pQ expansion increase dynamics and aggregation propensity in these domains.

The behavior of pQe‐AR observed here therefore aligns with previous reports on polyglutamine expanded proteins and provides a foundational basis for a unifying mechanism for polyglutamine and other misfolding diseases. In these disorders, (toxic) monomeric proteins aggregate into insoluble fibers, a process initially thought to underlie pathogenicity (Merry et al., 1998). However, other studies have questioned whether aggregation is the primary cause of cytotoxicity (Sisodia, 1998). Could the formation of insoluble fibers be cytoprotective? Previous reports on Huntingtin (Htt) suggest that inclusion body formation reduces the diffusion of Htt protein, thereby decreasing neuronal cell death (Arrasate et al., 2004). Our model, where the FQNLF and other functional motifs are blocked through multimerization, may suggest a similar protective mechanism, limiting (aberrant) function. Oligomerization of pQe AR leads to aggregation into insoluble fibers, which, at least initially, may act as a cytoprotective mechanism.

## 4 CONCLUSIONS

This study presents the first molecular model of an expanded polyglutamine tract containing the NTD of AR, offering unprecedented insights into the structural dynamics and functional implications of pQ expansion. Using extensive in silico simulations, topological mapping, and molecular docking analyses, we have provided a detailed understanding of the complex intra‐ and interdomain interactions within AR‐NTD, highlighting the effects of pQe on its structural reorganization, interactions with other AR domains, and cofactor binding, particularly RAP74, HSP70, and CHIP.

Our findings reveal that the expansion of the pQ tract induces a global dynamic restructuring of the AR‐NTD, driven by long‐range interactions of the glutamine residues with residues in the CR region. This reorganization disrupts the native dynamic regional segregation seen in wt AR‐NTD. Importantly, these long‐range interactions seem to include the ANTS motif, which was previously reported to crosstalk with the pQ tract of AR in the control of aggregation (Oppong et al., 2017). Interestingly, that study demonstrated that fibrillar aggregation of NTD fragments only occurred with pQe tracts, suggesting that the distance between the glutamine tract and ANTS was of importance. Our pQe NTD model does provide topological information on molecular interactions between the pQe and the ANTS motif. In contrast, this interaction is not seen with the wt‐NTD. This solidifies the potential importance of this motif and the positive regulatory crosstalk these have.

Additionally, our model underscores the delicate balance of PPIs. While pQ expansion results in the disruption of critical interactions—such as those between AR and TFIIF through RAP74—leading to impaired gene regulation, it may simultaneously enhance DNA binding through the loss of self‐inhibitory interactions. Furthermore, our results provide atomistic details on oligomerization of pQe‐AR, a process that could represent a cytoprotective mechanism. However, this oligomerization disrupts key PPIs, including interaction with HSP70 and CHIP, which are essential for protein homeostasis. These alterations in PPIs as a result of pQe mediated dynamic misfolding can ultimately disrupt numerous cellular processes essential for cell survival, from homeostasis to gene expression. Dysregulation of these multiple pathways could culminate in toxicity in a cumulative fashion, suggesting that therapeutic targeting of a single pathway may not provide complete amelioration. In contrast, reports of reducing cellular levels of pQe AR have emerged as an appealing therapeutic strategy to target the proximal mediator of disease pathogenesis (Eftekharzadeh et al., 2019). In this regard, our model opens new therapeutic avenues by revealing the distinct interaction patterns between the pQe tract and AR motifs. Targeting these interactions could potentially stabilize or reverse the pathological effect of pQe‐AR. Further experiments will be necessary to gain a deeper understanding of the altered transcription and regulation through PPIs by AR‐NTD, for which our models can serve as a framework. Preliminary findings (Supporting Information) suggest that the interaction between the pQ tract and the LBD may further influence AR function by modulating the N/C interaction (He et al., 2000), which can be affected by neighboring motifs (Dubbink et al., 2004), Although current computational methodologies limit the study of interactions between disordered regions like NTD and folded domains like LBD, our initial peptide docking results provide intriguing evidence that the pQ tract interacting with a pocket on the LBD (Figure S8) could modulate the FQNLF/AF‐2 interaction through a conformational change (Figure S9). This binding pocket was previously identified as a highly conserved structural pocket, the BF‐3 (Buzón et al., 2012). It is possible that expansion of the pQ tract affects this process in SBMA.

In conclusion, despite the incredible complexity of modeling nuclear receptors and large intrinsically disordered proteins, our approach has generated atomistic‐level insights that are consistent with experimental observations. This work not only provides a detailed understanding of the structural dynamics of AR in the context of polyglutamine expansion but also sets the stage for future studies of other disordered proteins and their pathological mutations. By unlocking new mechanistic insights, this model paves the way for the development of novel therapeutic strategies aimed at mitigating the effects of misfolding and aggregation in neurodegenerative diseases.

## 5 METHODS

### 5.1 MD simulations of AR‐NTD

This study presented MD simulation data on wt and pQe‐NTD using SIRAH and a99SBdisp. All details of our simulations are depicted in Figure S12. Initial representations of pQe AR‐NTD were generated using I‐TASSER (Yang et al., 2015) and mapped to the coarse‐grained (CG) SIRAH force field. The CG models were solved in octahedron boxes with a minimum distance of 1.2 nm from the solute using pre‐equilibrated WT4 water molecules. The ionic strength was set to 0.15 M by randomly replacing WT4 molecules with Na^+^ and Cl^−^ CG ions. The system was prepared following standard SIRAH protocols. First, energy minimization was performed with positional restraints of 1000 kJ mol^−1^ nm^−2^, followed by a 100 ps NVT simulation at 300 K and a 100 ps NPT equilibration. Production simulations were performed in the NPT ensemble at 310 K and 1 bar using GROMACS 2020 (Abraham et al., 2015) with a time step of 20 fs. PME electrostatics were used with the Verlet scheme and a cut‐off of 1.2 nm. Solvent and solute were coupled separately to velocity rescale thermostats with coupling times of 2 ps. Pressure was controlled by the Parrinello–Rahman barostat with a coupling time of 10 ps. Production trajectories were generated for 5 μs.

### 5.2 Initial structure preparation for all‐atom simulations

Initial structures for all‐atom simulations were generated from the I‐TASSER structure. Initially, structures were subjected to pulling via steered MD simulations to form a fully extended chain. Subsequently, 50 ns all‐atom simulation in implicit solvent was conducted to produce new initial structures. The QuickMD module of visual molecular dynamics (VMD) (Humphrey et al., 1996) in conjunction with the NAMD MD package (Phillips et al., 2020).

### 5.3 All‐atom MD simulations

All‐atom simulations on wt‐NTD, pQe‐NTD, and isolated pQ tracts (Q23 and Q45) were performed using a99SBdisp forcefield with compatible amber water molecules. A total of 1000 ns were simulated with a 2 fs time step. Neighbor searching was performed every 10 steps. The PME algorithm was used for electrostatic interactions with a 1 nm cut‐off. A single cut‐off of 1.006 nm was used for Van der Waals interactions. Temperature coupling was done with the V‐rescale algorithm, and Pressure coupling was done with the Parrinello‐Rahman algorithm. Trajectory analysis and visualization for all simulations were performed using VMD and GROMACS tools.

### 5.4 Clustering

To consider the high dynamicity of the disordered chain, we combined the dynamic phases of our simulations (the phase of the simulation when the chain reached the plateau in the RMSD trajectory), which corresponds to the last 800 ns of the all‐atom simulations and the last 3.5 μs of our coarse‐grained simulations. We clustered these combined MD trajectories with the GROMOS clustering algorithm in the GROMACS 2020 gmx cluster module. The RMSD cutoff value for the clusters was set to 0.9 nm.

### 5.5 Circuit topology analysis

CT parameters were analyzed from the final 850 ns of each aa99SBdisp simulation using custom Python scripts (Moes et al., 2022; Scalvini et al., 2023). These scripts were modified to include energy and length filtering, as well as circuit decomposition. Residue contacts were defined by a 4.5 Å distance cutoff, requiring at least five atoms from each residue to be within this distance. To avoid local overcounting, the three nearest neighbors of each residue were excluded from the contact analysis.

Residue‐based local CT was extracted using MATLAB based on established methods (Moes et al., 2022; Woodard et al., 2022). A cutoff scheme of 5 or more atom‐atom contacts within 4.5 Angstroms was employed, excluding contacts within 3 residues along the protein sequence. CT was calculated over the final 700 ns of the all‐atom simulations, at a 25 ns timestep, for all three simulations. Error was calculated as SEM over all extracted frames.

### 5.6 AR‐NTD protein–protein interactions

The atomic coordinates of the pQe‐NTD and wt‐NTD were taken from our structural SIRAH and aa99SBdisp models and used as input for PPIs using biased rigid body docking with ClusPro 2.0 webserver (Kozakov et al., 2017). The atomic coordinates of partner proteins (AR‐DBD: PDB 1R4I; AR‐LBD: PDB 1T7T; CHIP: residue 311–369 extracted from AlphaFold ID P50502; RAP74 C‐terminal domain: PDB 1I27; HSP70: 4PO2) were selected as ligands. For multimeric docking, the atomic coordinates of our NTD models were taken as both receptor and ligand.

For the inputs of ClusPro, we took the number of clusters that cover at least 50% of the poses. ClusPro predicted up to 120 different poses per protein–protein combination. All poses predicted by ClusPro were inspected using VMD and analyzed using a custom‐made Python script, utilizing different libraries: madplotlib (Hunter, 2007), mdanalysis (Alibay et al., 2023; Michaud‐Agrawal et al., 2011; Naughton et al., 2022), mdtraj (McGibbon et al., 2015), numpy (Harris et al., 2020), pandas (McKinney, 2010), scipy (Virtanen et al., 2020), and seaborn (Waskom, 2021).

## AUTHOR CONTRIBUTIONS

**Laurens W. H. J. Heling:** Investigation; visualization; formal analysis; data curation; writing – original draft; writing – review and editing; validation. **Vahid Sheikhhassani:** Methodology; investigation; formal analysis; data curation; writing – original draft; writing – review and editing. **Julian Ng:** Investigation; writing – review and editing; funding acquisition. **Morris van Vliet:** Investigation. **Alba Jiménez‐Panizo:** Investigation; writing – review and editing. **Andrea Alegre‐Martí:** Investigation; writing – review and editing. **Jaie Woodard:** Investigation; writing – review and editing. **Willeke van Roon‐Mom:** Writing – review and editing. **Iain J. McEwan:** Writing – review and editing; resources; investigation. **Eva Estébanez‐Perpiñá:** Investigation; writing – review and editing; resources. **Alireza Mashaghi:** Conceptualization; investigation; resources; supervision; project administration; writing – review and editing; writing – original draft; funding acquisition.

## CONFLICT OF INTEREST STATEMENT

The authors have no conflicts of interest to disclose.

## Supporting information


**Data S1.** Supporting Information.

## Data Availability

The raw simulation data generated during this study have been deposited in the following Zenodo repository: DOI 10.5281/zenodo.14512908
